# Alleviating a Nomad's Anguish: Successful Treatment of a Case of Leg Mycetoma—A Case Report

**DOI:** 10.1155/2012/753174

**Published:** 2012-12-03

**Authors:** Anthony Muchiri Maina, Joseph Theuri Macharia

**Affiliations:** ^1^Orthopaedic Department, AIC Kijabe Hospital, 00220 Kijabe, Kenya; ^2^AIC CURE International Children's Hospital, 00220 Kijabe, Kenya

## Abstract

*Introduction*. Mycetoma is a localized, chronic, progressive, granulomatous, inflammatory, non contagious, tumour-like lesion with sinuses discharging different types of granules. The organisms are usually inoculated into any body part subject to trauma-usually the foot. Treatment is medical and/or surgical. Prognosis is good in early cases with high recurrences in late cases or those inadequately treated. The authors describe the successful treatment of a severe case of leg mycetoma, with combined surgical and medical therapies.

## 1. Introduction

Mycetoma is also called maduromycosis, kirinagra, and Madura foot [1]. It is a clinical entity characterized by a chronic, localized, progressive, granulomatous, inflammatory, noncontagious, tumour-like lesion, common in the tropics and subtropics [2]. The highest incidence is in Sudan and it is 4 times more common in males, most often in farmers and field workers who are frequently exposed to minor penetrating wounds by thorns and splinters [3]. 75% of the lesions occur in the lower limbs [3]. It usually involves the foot especially in those who walk barefoot. The trauma results in the classic triad tumefaction, multiple sinuses discharging different types of granules. The grains are an aggregation of organisms, either fungi (Eumycetes) Schizomycetes (gram-positive filamentous bacteria, true bacteria {botryomycosis}), or in combination. Diagnosis entails clinical exam, high index of suspicion, and isolation of the organism [4]. 

## 2. Case Presentation

**Figure 1:**
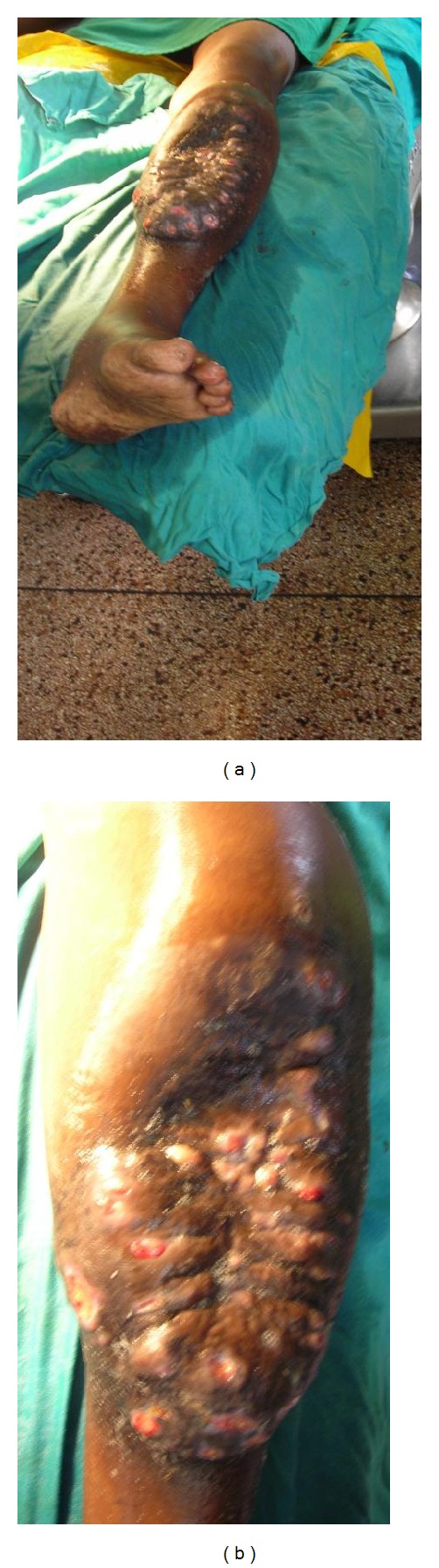
(a) Gross view of left leg anteromedial swelling sparing the knee, distal leg, ankle, and foot; (b) Closer view of the swelling showing the pouting sinuses.

**Figure 2:**
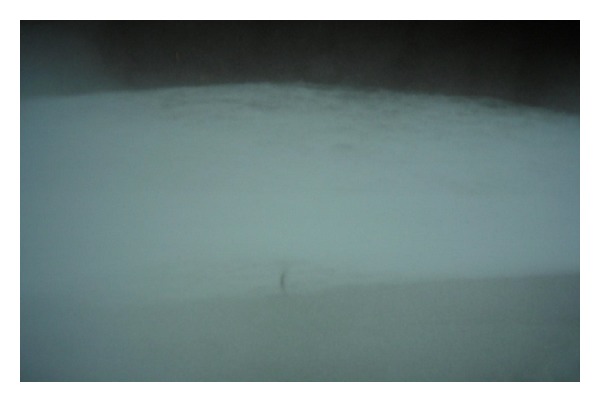
Lateral X-ray of the left tibia-fibula showing cortical lytic defects with no periosteal reaction (‘‘moth-eaten” appearance).

**Figure 3:**
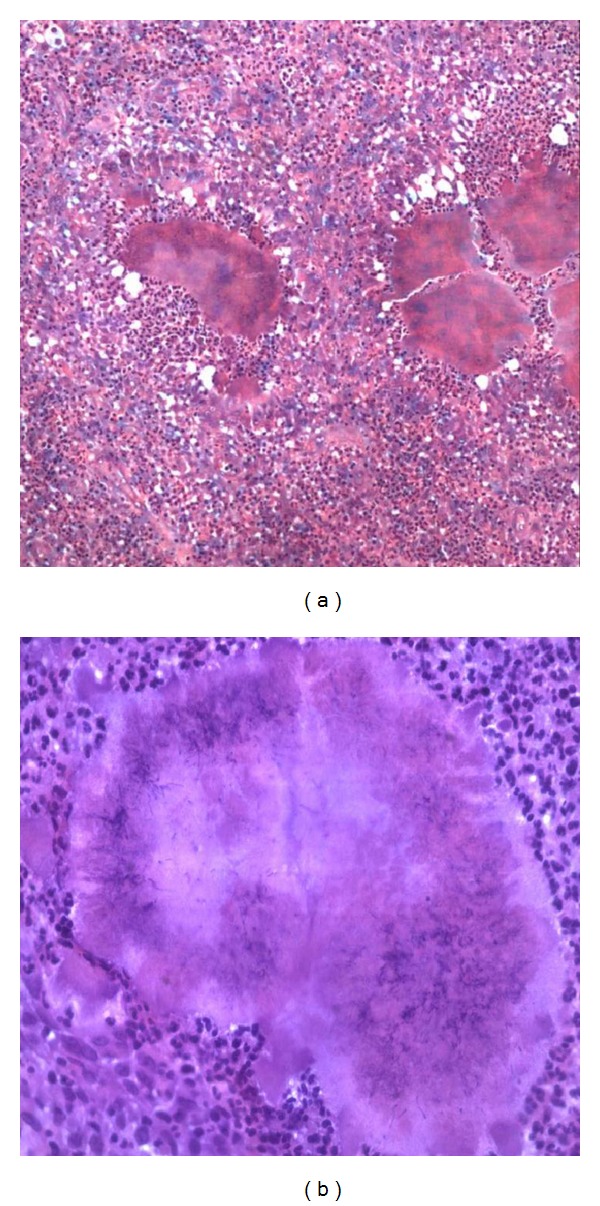
(a) Slide showing several clusters of mycetoma organisms surrounded by reactive zone of leucocytes (granulomas) ×10, H&E stain. (b) Closer look at a mycetoma granuloma showing the clusters of the organisms centrally and the surrounding leucocytes ×20, H&E stain.

**Figure 4:**
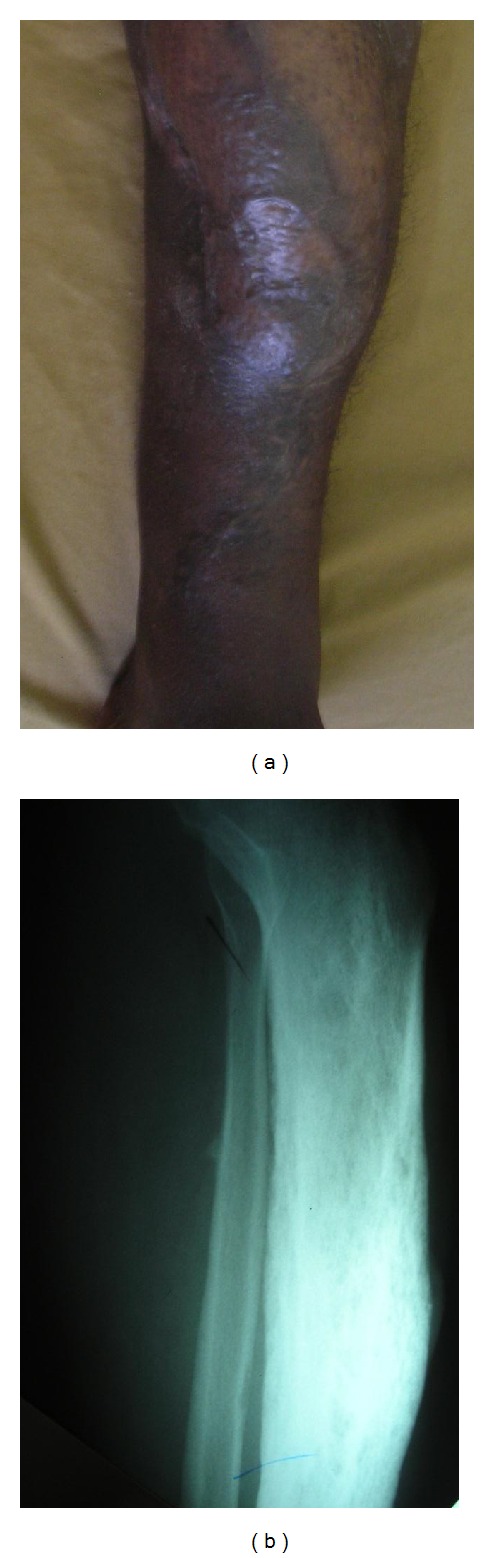
(a) shows the good take of the skin graft and gastrocsoleus flaps; the sinuses have not recurred. (b) Lateral X-ray of the left tibia-fibula showing the resolution of the lytic defects in the tibia.

A 34-year-old nomadic patient presented to the AIC Kijabe Hospital with a left leg anteromedial swelling associated with multiple sinuses discharging yellowish granules which had developed over the previous 4 years (Figures 1(a) and 1(b)). He denied any preceding trauma. He also had lost weight over the same period. There was pain localized to the lesion and progressive inability to use the limb. The foot and ankle had normal function. He also had right testicular pain that was successfully treated as epididymo-orchitis with ciprofloxacin. Radiographs revealed tibial superficial cortical lytic defects without periosteal reaction (Figure 2). He had a normocytic, normochromic anemia (5.4 g/dL) that warranted blood transfusion (10.9 g/dL) before surgery and a leucocytosis (15,500/mm^3^). The HIV test was negative. An incisional biopsy revealed both actinomycetoma and eumycetoma with abscesses (Figures 3(a) and 3(b)). Definitively, excision was done with gastrocsoleus flaps and STSG filling the defect. Pre- and postoperatively he was treated with itraconazole and ciprofloxacin initially for 2.5 months then itraconazole and penicillin V for 8 months, with complete resolution (Figures 4(a) and 4(b)). After 2.5 months of treatment, the leukocytosis had resolved (7,400/mm^3^) and the patient was ambulant full weight-bearing. No signs of recurrence have been noted to date.

## 3. Discussion

Mycetoma is invasive and crippling and recurrence rates after treatment are high [4]. The duration of lesions vary from a few months to years and a history of a thorn prick or trauma is available in about 25% of cases [4]. All mycetoma agents have a similar clinical appearance, but atypical presentation in 10–15%, for example, cystic mass [1]. Thus, the diagnosis is mainly clinical and identification of the specific organism is the only further task. Bacterial mycetoma can usually be managed effectively with antibacterials alone (50–80% cure rates), while infections with fungi require antifungal medication and surgery [2, 4]. Without proper treatment, mycetoma can lead to deformity, amputation, and death [2]. There is no consensus on treatment regimes that are often prolonged with numerous relapses [4]. In eumycetoma, ketoconazole or itraconazole and surgery are the regime of choice [5, 6]. For actinomycetoma, various antibiotics have been used with good reported outcomes—Septrin, dapsone, amikacin, and penicillin V. Our patient who had both Eumycetes and actinomycetes as causative agents was treated with surgery (excision) and antimicrobials (itraconazole, ciprofloxacin and penicillin V). Ketoconazole is the treatment of choice for *Madurella mycetomatis* (most common cause of eumycetoma in hot areas) [6]. The choice of itraconazole was due to the higher likelihood of liver toxicity with ketoconazole.

## 4. Conclusion

This patient presented with a solitary leg mycetoma that was in Stage C (Table 1). The previous cases at our hospital with the same stage ended up with amputations due to their diffuse nature. Therefore, the localized (albeit deep) nature mitigated against an amputation. Moreover, the presence of a functional foot and the necessary medicosurgical armamentarium saved the day. Thus, as much it presents with invasion into deeper tissues in the late stages, amputation should not always be the first choice; treatment should be individualized and guided by the causative organism [5, 6], stage of disease, and whether diffuse or localized/solitary lesion(s). This paper presents a late stage of leg mycetoma that was successfully treated with limb and function preservation.

## Figures and Tables

**Table 1:** Staging-classification of mycetoma: a continuum of worsening disease from Stage A to D.

Stage	Description
A	Swelling, no sinuses
B	Woody induration with pustules/sinuses
C	Bone involvement on X-ray
D	Spread to other sites/multiple lesions

## Abbreviations

AIC: African Inland Church
HIV: Human immunodeficiency virus
STSG: Split-thickness skin graft.
